# Limited changes in spinal lamina I dorsal horn neurons following the cytotoxic ablation of non-peptidergic C-fibers

**DOI:** 10.1186/s12990-015-0060-z

**Published:** 2015-09-09

**Authors:** Abeer W. Saeed, Sophie A. Pawlowski, Alfredo Ribeiro-da-Silva

**Affiliations:** Department of Pharmacology and Therapeutics, McGill University, 3655 Promenade Sir-William-Osler, Montreal, QC H3G 1Y6 Canada; Alan Edwards Centre for Research on Pain, McGill University, Montreal, QC H3A 0G1 Canada; Department of Anatomy and Cell Biology, McGill University, Montreal, QC H3A 0C7 Canada

**Keywords:** IB4-saporin, NK-1 receptors, Rat, Behavior testing, Projection neurons

## Abstract

**Background:**

Non-peptidergic nociceptive neurons are a sub-population of small diameter primary sensory neurons that comprise approximately 50 % of the C fiber population. Together with the peptidergic sub-population, they transmit nociceptive information from the periphery to the superficial dorsal horn of the spinal cord. Despite the numerous studies investigating the role of the non-peptidergic primary afferents, their role in normal nociception and in pain remains poorly understood. Our lab has previously demonstrated that, in rat models of neuropathic and inflammatory pain, there is a de novo expression of substance P receptors (NK-1r) by lamina I pyramidal projection neurons, a neuronal population that normally does not express these receptors.

**Results:**

In this study, we used a ribosomal toxin, saporin, conjugated to the lectin IB4 to selectively ablate the non-peptidergic nociceptive C fibers, to investigate if the loss of these fibers was enough to induce a change in NK-1r expression by lamina I projection neurons. IB4-saporin treatment led to the permanent ablation of the IB4-positive afferents but also to a small non-significant reduction in CGRP-positive afferents. An overall increase in immunoreactivity for the NK-1r was observed in lamina I projection neurons, however, the lack of non-peptidergic afferents did not increase the number of lamina I pyramidal projection neurons immunoreactive for the receptor.

**Conclusions:**

Our results demonstrate that the deletion of the non-peptidergic afferents, at the L4–L5 spinal levels, is not sufficient to trigger the de novo expression of NK-1r by projection pyramidal neurons but increases the expression of NK-1r in fusiform and multipolar projection neurons. Furthermore, our data suggest that a neuropathic component is essential to trigger the expression of NK-1r by pyramidal neurons.

## Background

Most of the pain-related (nociceptive) information is relayed from the periphery to the spinal dorsal horn via small diameter primary afferents, which represent either unmyelinated (C) or thinly myelinated (Aδ) axons, with only a minority being of larger diameter (for reviews see [1, 2]). Usually, the smaller diameter nociceptive afferents are classified into two mostly independent subpopulations, the peptidergic and non-peptidergic [3, 4]. The peptidergic fibers express substance P (SP) and calcitonin gene-related peptide (CGRP) and depend on nerve growth factor for survival postnatally, while the non-peptidergic fibers are devoid of neuropeptides, bind the *Griffonia simplicifolia* isolectin-B4 (IB4), express the purinergic receptor P2X3 and depend on glial-derived neurotrophic factor for post-natal survival [5–7]. These populations also differ in their central termination in the spinal cord. Indeed, the peptidergic afferents terminate mostly in lamina I and outer lamina II, while the non-peptidergic project mostly to inner lamina II [1, 8–10]. A considerable number of peptidergic afferents, as well as a few non-peptidergic afferents terminate in contact with lamina I projection neurons [11, 12].

Lamina I projection neurons comprise three populations; fusiform, multipolar and pyramidal, which have distinctive morphology [13–16] and, possibly, function [17]. Fusiform neurons have an elongated soma with two dendrites at each end. Physiologically, in the cat, they have been shown to be nociceptive specific (NS), and respond to noxious heat and pinch [17]. Multipolar neurons have four or more dendrites arising from an irregularly-shaped soma and have been shown to be either NS or HPC (polymodal neurons responding to a variety of stimuli, including noxious heat, pinch and noxious and innocuous cold) [17]. In horizontal sections, pyramidal neurons have a triangular-shaped soma with one dendrite at each of the three tips. These neurons do not respond to noxious stimuli, however, they respond to innocuous cooling (COOL cells) [17]. While fusiform and multipolar neurons express the SP receptor (NK-1r) in rat and primate, most pyramidal neurons do not [13, 14]. This difference in NK-1r expression might provide a basis for why fusiform and multipolar neurons are nociceptive, whereas most pyramidal neurons might be non-nociceptive under physiological conditions [13, 14, 18].

The correlation between morphology and function of lamina I projection neurons is not accepted by all investigators. In rat, a majority of spinoparabrachial neurons were shown to respond to noxious stimuli [19, 20], and a high percentage of lamina I pyramidal projection neurons were considered NK-1r immunoreactive by another group [11, 21, 22]. However, our laboratory has consistently found that in naïve rats only about 22 % of the pyramidal neurons express the NK-1r [8, 23]. Interestingly, we found that the percentage of pyramidal neurons expressing the NK-1r increased drastically in animal models of neuropathic and arthritis pain [23, 24]. Furthermore, there was a significant increase in SP-immunoreactive innervation onto these neurons [23, 24]. These results indicate a phenotypic switch in pyramidal projection neurons in models of pain. What precipitates this phenomenon remains unknown.

In a chronic constriction injury model, a long-term loss of non-peptidergic afferents was reported both peripherally [25] and centrally [26, 27]. Because we have recently shown that lamina I projection neurons receive innervation from non-peptidergic afferents [12], it is possible that it is simply the loss of these fibers that triggers the upregulation of NK-1r on pyramidal neurons after nerve injury. To investigate this possibility, we used IB4 conjugated to the ribosomal toxin saporin, which when injected into the sciatic nerve leads to a permanent ablation of the non-peptidergic C fibers, mostly sparing the other fibers populations [28, 29]. Our main objective was to investigate whether the cytotoxic ablation of IB4-binding afferents was sufficient to induce a de novo expression of NK-1r by pyramidal neurons in otherwise naive rats or whether the increase in NK-1r observed in those animals was a result of increased immunoreactivity in the neurons that normally express it. Our working hypothesis was that a de novo expression of NK-1 receptors by lamina I pyramidal neurons should be partially triggered by the loss of the non-peptidergic C-fiber population. Ideally, we would have liked to suppress the peptidergic afferents as well, but there is not yet any specific way of ablating this population in rats. Our results suggest that the loss of non-peptidergic afferents does not play a significant role in the phenotypic switch of lamina I pyramidal neurons that we observed previously in a model of neuropathic pain [23].

## Results

### Behavioral assessment following injection of IB4-saporin

**Fig. 1:**
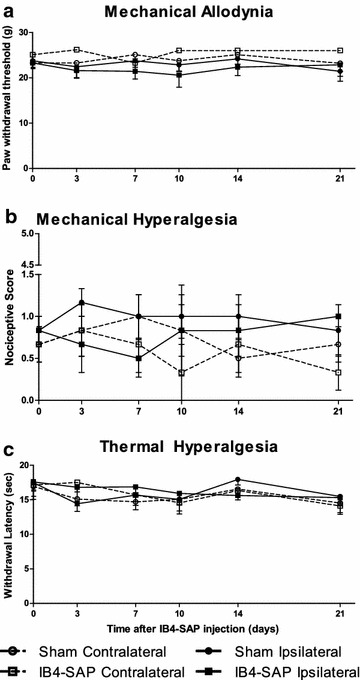
Behavioral characterization at several time points following IB4-saporin injection. **a** Assessment of mechanical allodynia using von Frey filaments in IB4-saporin-treated and sham animals; mechanical thresholds were not significantly different from the sham group. **b** Assessment of mechanical hyperalgesia using pin prick in IB4-saporin-injected and sham groups; there was no significant difference in mechanical nociceptive rating between IB4-saporin-injected and sham groups. **c** Assessment of thermal hyperalgesia using the Hargreaves test in IB4-saporin-injected and sham groups; IB4-saporin-injected animals did not display a significant difference in withdrawal latencies at any of the time points studied following IB4-saporin injection when compared with the sham group. N = 6, two-way ANOVA with Bonferroni post hoc

Animals injected with IB4-saporin into the sciatic nerve did not display any changes in evoked thresholds to the application of von Frey hairs (Fig. 1a), to an acute noxious stimulus (pin prick) (Fig. 1b) or to noxious thermal stimuli (Fig. 1c) up to 21 days following IB4-saporin injection when compared to vehicle-injected sham animals.

There was no difference from baseline in the contralateral side, either in IB4-saporin-injected or vehicle-injected groups (Fig. 1).

### Changes in spinal dorsal horn following IB4-saporin injection

**Fig. 2:**
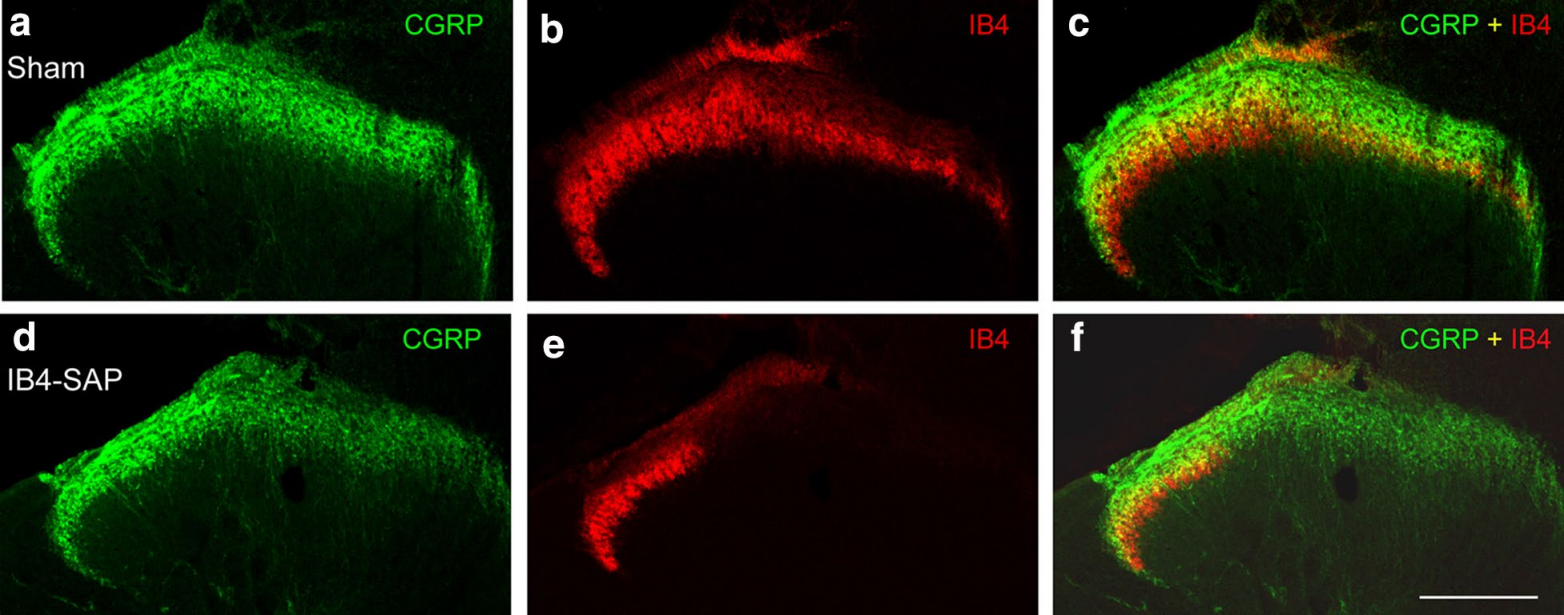
Confocal low magnification images from transverse sections comparing the ipsilateral side of the dorsal horn of vehicle-injected animals (**a**–**c**) with the ipsilateral side of IB4-saporin-injected (**d**–**f**) rats, at 21 days post-injection. In the *lower panels*, note the virtually complete depletion of IB4 staining (in *red*), except in the lateral third of the dorsal horn, that does not receive sciatic afferents. It is important to note that although IB4-saporin binds to non-peptidergic fibers, a slight reduction of CGRP immunoreactivity (in *green*) in the area of the lesion was noticeable when compared to sham. *Scale bar* 100 µm

**Fig. 3:**
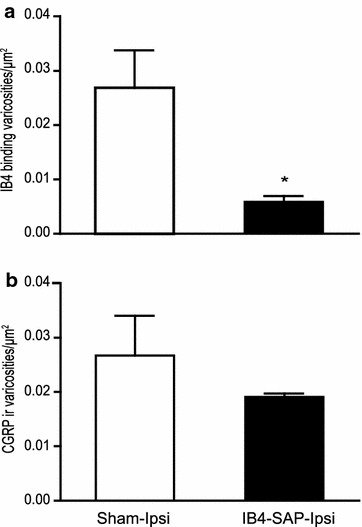
Quantitative analysis of the density of IB4-binding (**a**) and CGRP-ir (**b**) varicosities per area unit (µm^2^) in the sciatic territory, 21 days after injection in the sciatic nerve of IB4-saporin (IB4-SAP, N = 5) or saline (sham, N = 4), in the ipsilateral dorsal horn. **a** Ipsilateral to IB4-saporin injection the number of IB4-positive varicosities was reduced (IB4-SAP) compared to sham animals (Student’s t-test; *P < 0.02). **b** Ipsilateral to IB4-saporin injection, the density of CGRP ir varicosities was slightly reduced, but the difference from sham was not significant (Student’s t-test)

At 3 weeks after the injection of IB4-saporin into the sciatic nerve, we detected a virtually complete loss of non-peptidergic afferents on the side ipsilateral to the injection at the level of the L4–L5 spinal segments compared to shams which had no loss (Fig. 2). The area of loss of staining corresponded to the medial two-thirds of the dorsal horn. The labeling persisting in the lateral third corresponded to non-sciatic afferents. Quantitative analysis of IB4-positive varicosities (lamina II), 21 days after injection in the sciatic nerve, supported this observation (Fig. 3a). The density of IB4-positive varicosities ipsilateral to IB4-SAP injection decreased significantly compared to sham animals (Fig. 3a). We also detected a small (Fig. 2) but non-significant loss of CGRP-immunoreactive (ir) varicosities in laminae I–II in IB4-SAP injected rats (Fig. 3b) compared to sham animals. There were no contralateral changes in either IB4 or CGRP labeling (Fig. 3).

**Fig. 4:**
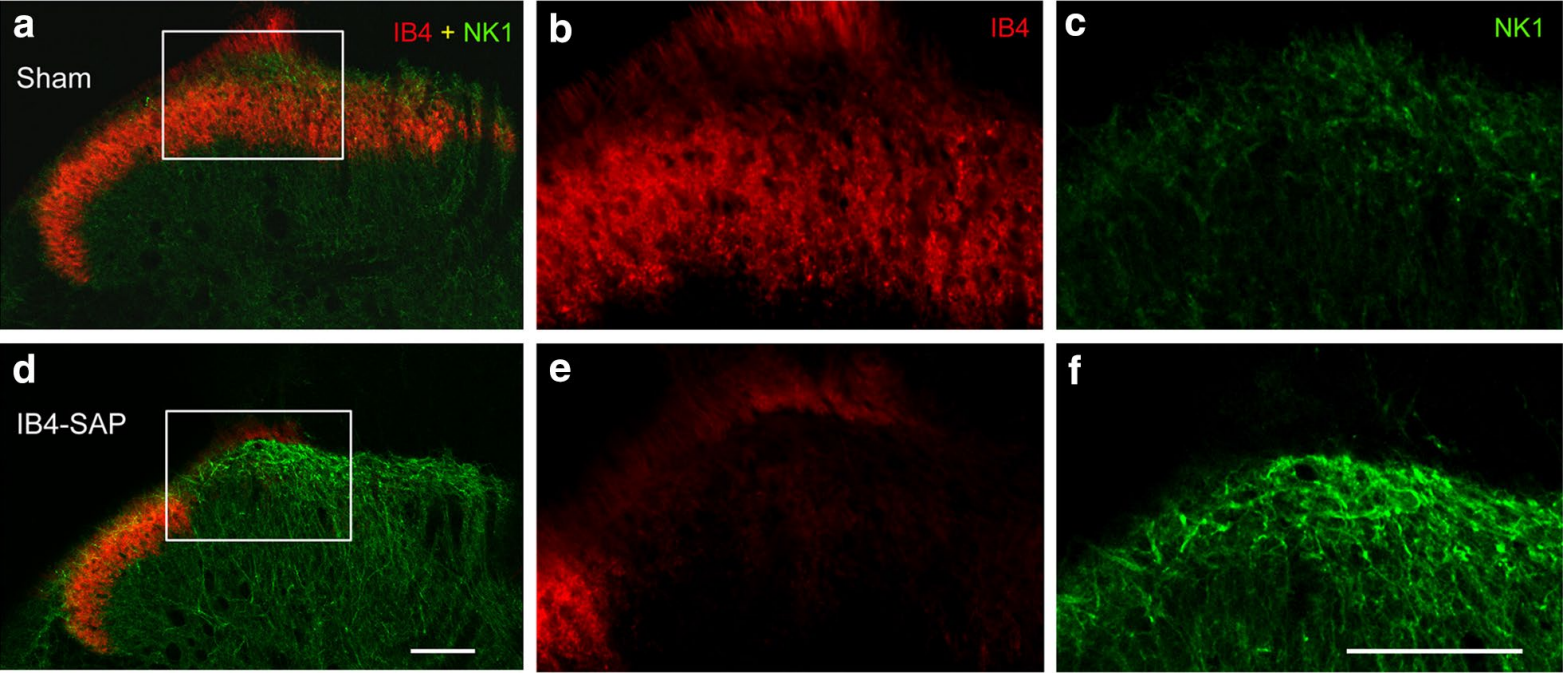
Confocal images comparing NK-1r immunoreactivity in the ipsilateral side of vehicle-injected (**a**–**c**) and IB4-saporin-injected (**d**–**f**) animals, in transverse sections, 21 days after injection. NK-1 receptors—NK-1 (in *green*), non-peptidergic afferents—IB4 (in *red*). The framed regions in **a** and **d** are enlarged in **b**, **c**, **e** and **f**. *Scale bars* 100 µm

Interestingly, we observed an increase in NK-1r immunoreactivity in lamina I in IB4-saporin-treated rats when compared to sham animals (Fig. 4).

### Observations in lamina I spinoparabrachial neurons

**Fig. 5:**
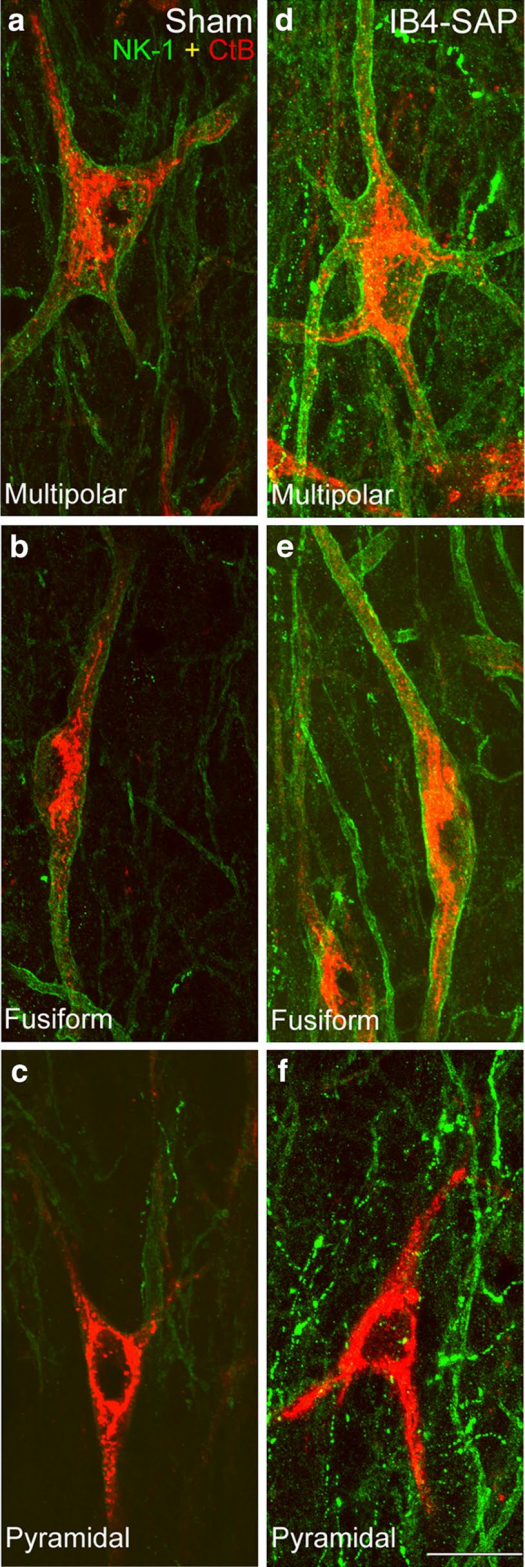
Confocal images at high magnification showing the morphology and NK-1r expression by the lamina I spinoparabrachial neuronal population in IB4-saporin-injected animals compared to the vehicle-injected sham group at the 21 days time point. Both fusiform and multipolar neurons showed an increase in NK-1r immunoreactivity in each cell (**d**, **e**), compared to the sham group (**a**, **b**). However, the great majority of pyramidal neurons did not display NK-1r immunoreactivity following IB4-saporin injection at the same time point (**c**, **f**). NK-1 receptor—NK-1 (in *green*), retrograde tracer CTb (in *red*). *Scale bar* 20 µm

As in previous publications from our laboratory, spinoparabrachial lamina I neuronal populations were identified based on the dendritic arborization and cell body shape as viewed in the horizontal plane. Multipolar neurons possess four or more primary dendrites arising from an irregularly-shaped cell body (Fig. 5a). Fusiform neurons have one dendrite arising from each end of an elongated soma (Fig. 5b). Pyramidal neurons have a triangularly-shaped soma with a primary dendrite originating from each of the three corners (Fig. 5c). Some cells displayed features transitional between two of the cell types described above or had a portion of the cell body and/or proximal dendritic tree truncated due to sectioning. These latter neurons were not classifiable and were considered as “unclassified”.

**Fig. 6:**
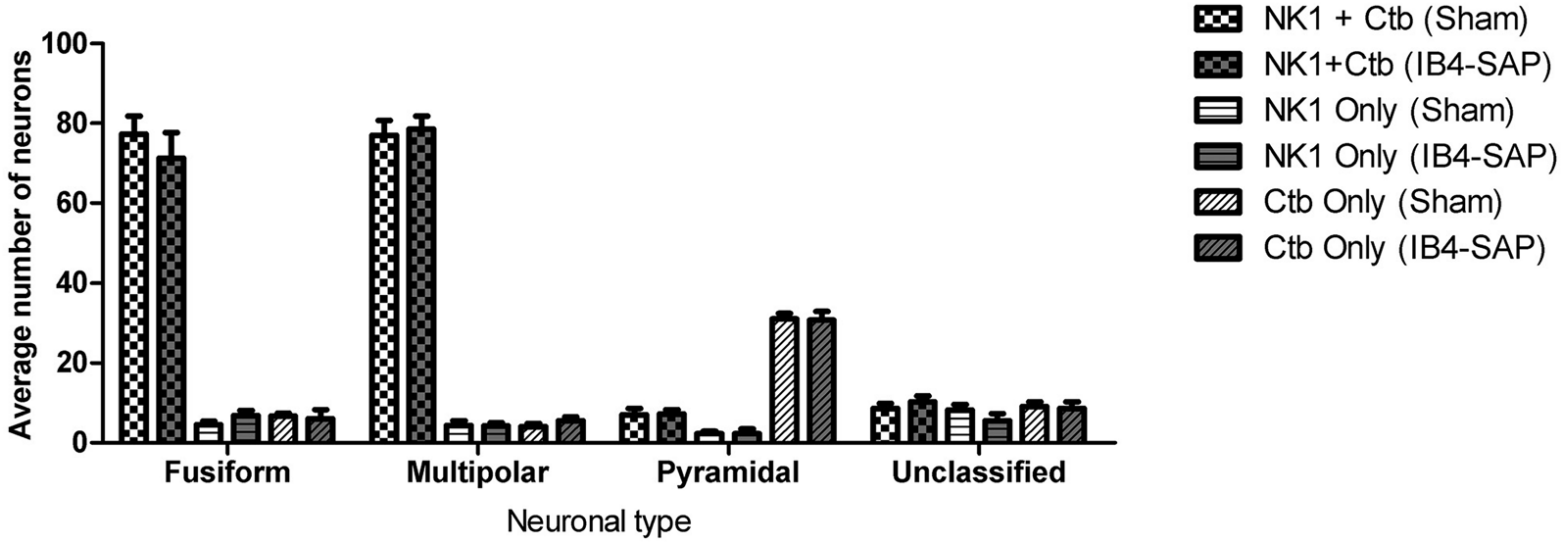
Quantitative comparison of the lamina I neuronal populations immunoreactive for the NK-1r (NK-1 only), retrogradely labeled from the parabrachial nucleus (CTb only) or double labeled (NK-1 + CTb), on the ipsilateral side of IB4-saporin-injected or sham group at 21 days. There were no changes in the number of fusiform, multipolar, or pyramidal neurons immunoreactive for one or both of the markers when comparing IB4-saporin-injected to sham group. Only cells with visible nuclei were counted. Values represent average number of neurons (±SEM) counted per animal. N = 6, one-way ANOVA

In sham animals, we confirmed our previous observations that spinoparabrachial multipolar and fusiform neurons displayed immunoreactivity for NK-1r in a high proportion of cells, whereas pyramidal neurons were almost never immunoreactive for the receptor (Figs. 5, 6). We observed an increase in the intensity of NK-1r staining in multipolar and fusiform neurons, when comparing the ipsilateral side of the spinal dorsal horn of IB4-saporin-injected animals (Fig. 5d, e) to the ipsilateral side of vehicle-injected sham animals (Fig. 5a, b), although there was no change in the proportion of neurons expressing immunoreactivity for the receptor (Fig. 6). Surprisingly, the number of pyramidal neurons expressing detectable NK-1r immunoreactivity remained very low (Fig. 6).

## Discussion

In this study, we observed that the selective ablation of non-peptidergic nociceptive primary afferents by means of IB4-saporin injection into the sciatic nerve did not cause any change in pain-related behavior compared to sham animals. This raises the possibility that normal behavioral function was maintained either due to redundancy in the IB4 afferent population or due to a compensatory mechanism in the spinal cord. We detected an overall increase in the expression of NK-1r in populations of lamina I projection neurons that already expressed the NK-1r, which would support the idea of spinal compensatory mechanisms. Nevertheless, we did not observe any de novo expression of NK-1r by pyramidal neurons or any change in the total number of lamina I neurons that expressed the NK-1r, in agreement with the absence of behavior changes.

### Effect of IB4-saporin injection on non-peptidergic primary sensory neurons

When injected in a peripheral nerve, IB4-saporin is known to cause a selective loss of primary afferents binding IB4 [28, 29]. The lectin IB4 from the conjugate binds selectively to the α-d-galactoside of versican, an extracellular matrix protein expressed only on the non-peptidergic afferents and a small number of peptidergic afferents. IB4-saporin is retrogradely transported to the dorsal root ganglia, where the saporin component induces cell death through mitochondrial toxicity. Several studies agree that the maximal loss of IB4 binding occurs by 21 days after IB4-saporin injection and that this loss persists indefinitely as it results from cell death [28, 29]. In addition to a decrease in number of IB4-positive varicosities in lamina II, we observed a small, non-significant decrease in the density of CGRP-ir peptidergic varicosities in laminae I–II. This result might be correlated with studies that highlighted the binding of IB4 on peptidergic afferents [7, 30]. This result is in agreement with the slight decrease in SP or CGRP immunoreactivity following IB4-saporin treatment reported by previous studies [28, 29, 31].

### Behavioral changes following IB4-saporin injection

Consistent with previous studies [28, 29], we did not detect any changes in the pain thresholds for innocuous mechanical or noxious mechanical and thermal stimuli 21 days after IB4-saporin injection. A previous study from our laboratory in which the IB4-saporin was injected bilaterally into the mental nerves did not reveal any difference from controls when mechanical thresholds were tested in the lower lip skin [31]. These results are in agreement with previous work that describes the lack of behavioral changes following ablation of IB4-positive epidermal innervations [32]. On the other hand, our results at earlier time points differ from those of groups [28, 29] who previously injected IB4-saporin into the sciatic nerve of rats and reported a transient elevation in mechanical and thermal thresholds at early time points (up to day 14 after IB4-saporin injection).

This lack of mechanical and thermal changes following the complete ablation of non-peptidergic afferents after an intrasciatic IB4-saporin injection, may indicate a redundancy in the unmyelinated C fiber populations. In the rat, the peptidergic and non-peptidergic afferents both express the vanilloid receptor TRPV1 [33], which suggests that these two C fiber populations overlap in nociceptive function, unlike studies in the mouse, which advocate specific modalities for each of the two C fiber populations [34, 35]. Therefore, it is possible that even if they are slightly affected, the peptidergic afferents are able to compensate for the loss of the non-peptidergic afferents.

### Changes in dorsal horn lamina I projection neurons following IB4-saporin injection

Following the loss of non-peptidergic afferents after the intrasciatic injection of IB4-saporin, we observed an overall increase in NK-1r immunoreactivity by dorsal horn lamina I projection neurons (Fig. 4). Previous studies demonstrated an increased NK-1r immunoreactivity associated with a novel expression of the receptor by lamina I pyramidal neurons in pain models of inflammation [24] and neuropathy [23]. This increase suggested a compensatory mechanism at the level of the spinal cord, however, our quantitative study of NK-1r expression by the three lamina I projection neuronal populations did not reveal any novel expression of NK-1r by pyramidal neurons or an increased number of cells of the fusiform or multipolar neuronal populations (Fig. 6). This indicated that the increased NK-1r immunoreactivity we detected was caused by an increase in the number of receptors by cell populations already expressing it. These results were expected since the de novo expression of NK-1r by pyramidal neurons seems to correlate with a chronic pain state, whereby the nociceptive system is being stimulated following an injury, which was not the case in this study.

Work by Taylor et al. [31] in a model of trigeminal neuropathic pain demonstrated an increased hypersensitivity to mechanical stimuli when the neuropathic injury is preceded by an IB4-saporin injection, however these results should be confirmed in a sciatic nerve neuropathic pain model.

## Conclusions

In this study, we have demonstrated that the lesioning of the non-peptidergic afferents of the sciatic nerve does not cause any significant pain-related behavior change for up to 21 days after lesion. In the spinal dorsal horn, changes were limited to the loss of the non-peptidergic afferents and a strong increase in overall immunoreactivity for the NK-1r, without a change in the overall number of projection neurons expressing the NK-1r. These results suggest that, in naïve rats, the IB4-saporin-induced loss of non-peptidergic afferents is not sufficient to trigger the NK-1r de novo expression by pyramidal neurons.

## Methods

The experimental procedures followed the guidelines for the Care and Use of Experimental Animals of the Canadian Council on Animal Care and the International Association for the Study of Pain (IASP). All protocols were approved by the McGill University Faculty of Medicine Animal Care Committee.

Forty male Sprague–Dawley rats (Charles River, QC, Canada), weighing between 220 and 230 g, were used. The number of animals used and their suffering was kept to the minimum necessary for the conduction of the study. Animals were exposed to 12 h light/dark cycles with food and water available ad libitum and were housed four animals to a cage fitted with soft bedding and a plastic tube for an enriched environment.

### Animal preparation

#### Surgeries

Animals were anesthetized with 5 % isoflurane in oxygen. Unilateral injections were carried out on the left sciatic nerve. Using blunt dissection, the left biceps femoris and gluteus superficialis muscles were separated. Care was taken to minimize the stretching of the sciatic nerve when it was separated from the surrounding connective tissue. Experimental animals received a total of 6 µL of an 800 μg/mL solution of IB4-saporin (Advanced Targeting Systems, San Diego CA, USA) in 0.2 M Phosphate Buffered Saline (PBS) and Fast Green Dye (Sigma, MO, USA) injected at three injection sites into the sciatic nerve proximal to its branching point using calibrated glass micropipettes (Wiretrol II, Drummond Scientific Company, Broomall, PA, USA). The Fast Green dye was used to monitor the accuracy of the injection. The control group was injected with vehicle solution of 6 µL 0.2 M PBS in Fast Green dye using the same method. The incision was sutured in two layers using 4-0 Vicryl sutures (Ethicon Inc, New Jersey, USA). Animals were returned to their cages to recover. No difference in weight gain between experimental and sham groups was observed at any time point throughout the study.

#### Injection of tracer

To retrogradely trace projection neurons, animals were first anesthetized using 5 % isoflurane in oxygen, placed in a stereotaxic apparatus (David Kopf Instruments, Tujunga, CA, USA) and the head stabilized with non-perforating ear bars. The coordinates for the parabrachial nucleus (rostral/caudal: −9.12 mm; medial/lateral: −2.1 mm; dorsal/ventral: −6.3 mm) were calculated, with Bregma as the reference point, from the Paxinos & Watson Rat Brain Atlas [36]. A small hole was drilled through the skull at the target point, exposing the dura mater. A glass micropipette (Wiretrol II, Drummond Scientific Company, Broomall, PA, USA) was lowered, through the dura mater, to the stereotaxic position of the parabrachial nucleus and 2 µL of 1.0 % solution of cholera toxin subunit B (CTb) (List, Campbell, CA, USA) was slowly injected over a period of 20 min. To minimize leakage of the tracer, a 10 min waiting period was imposed before the micropipette was retracted from its position. CTb was injected 7 days prior to sacrificing the animals.

### Behavior testing

Animals were always habituated to the testing apparatus for 30 min prior to testing. Baseline behavioral thresholds were measured for 2 consecutive days prior to surgical treatments.

#### Testing for mechanical allodynia

Animals were placed in clear plastic enclosures elevated on a mesh grid, which allowed complete access to the ventral side of the animal. Animals were tested using the up-down method previously described by Chaplan et al. [37]. Filaments of increasing stiffness were applied to the mid-plantar area of the hind paw, avoiding the foot pads, until an obvious withdrawal or flicking/licking behavior occurred. In case of a positive response, the next weaker filament was presented, however, in the case of a negative response; the next stronger filament was applied. This process was repeated until six responses were recorded and a mean threshold was calculated. The testing was performed on the right paw of all the rats followed by the testing of the left paw in the same manner.

#### Testing for mechanical hyperalgesia

Mechanical hyperalgesia was assessed using the pin prick method described by Coderre et al. [38]. The point of a blunted 23 gauge needle was applied to the skin of the heel (touching, but not penetrating). Behavioral responses to the pin prick were rated according to the following scale: 0 = no response; 1 = rapid paw flicking, stamping, or shaking (less than 1 s); 2 = repeated paw stamping, shaking, or paw lift less than 3 s; 3 = above behaviors or hind paw licking for more than 3 s; 4 = above behaviors for more than 3 s and hind paw licking for more than 3 s. An additional point was added if any vocalizations occurred. The mean for reaction for each paw was calculated.

#### Testing for thermal hyperalgesia

The Hargreaves test [39] was used to measure thermal nociceptive thresholds. Clear plastic enclosures were set on top of a glass floor. The light source was directed onto skin area of the paw in contact with the glass. The time from turning on of light source until withdrawal was noted. Testing included three trials per paw with each trial being completed for all the animals before the start of the next trial. This ensured there was a 30 min wait before the start of the next trial to minimize desensitization effects. The average of the three trials per paw was calculated.

### Animal perfusion

At the end of the experiment (21 days after the injection IB4-saporin or sham injection), the animals were deeply anesthetized with Equithesin (6.5 mg chloral hydrate and 3 mg sodium pentobarbital in a volume of 0.3 mL, i.p., per 100 g body weight). They were then perfused through the left cardiac ventricle with perfusion buffer (for composition see [40]) for 1 min, followed by 30 min of 4 % paraformaldehyde in 0.1 M phosphate buffer (PB), pH 7.4. The brain and spinal cord segments L4–L5 were extracted and postfixed for 4 and 2 h, respectively, in 4 % paraformaldehyde in PB. The specimens were then cryoprotected in 30 % sucrose in PB overnight at 4 °C.

### Immunohistochemistry

Serial 100 µm-thick coronal sections of the brain were obtained, to examine the site of injection at the level of the parabrachial nucleus. L4–L5 spinal cord segments were trimmed and cut serially at 50 µm thickness. Both horizontal and coronal sections were cut using a freezing-sledge microtome (Leica, Richmond Hill, ON, Canada). Sections were collected as free-floating in phosphate-buffered saline (PBS) with 0.2 % Triton-X 100 (PBS + T).

To examine lamina I projection neurons, horizontal sections were incubated in 10 % normal donkey serum (Jackson, West Grove, PA, USA) in PBS + T for 1 h at room temperature to block unspecific staining. Subsequently, spinal cord sections were incubated, for 48 h at 4 °C, with the primary antibodies: goat anti-CTb at 1:5000 dilution (List Biological), rabbit anti-NK-1r, raised against residues 376–407 of the C-terminal sequence of the rat receptor, at 1:10,000 dilution (Sigma) and IB4 conjugated to AlexaFluor 647 (Molecular Probes) in PBS + T containing 5 % normal donkey serum. Then, the sections were washed several times with PBS + T and incubated for 2 h at room temperature with secondary antibodies preabsorbed with 10 mg/mL acetone rat brain powder: donkey anti-goat Rhodamine Red X (1:200, Jackson, West Grove, PA, USA) and donkey anti-rabbit AlexaFluor 488 (Molecular Probes). Finally, sections were washed with PBS, mounted on gelatin-subbed slides and coverslipped with an anti-fading mounting medium (Aqua Polymount; Polysciences, Warrington, PA, USA). Slides were stored at −4 °C. Control sections were processed by omitting the primary antibody which resulted in complete loss of immunoreactivity. The above protocol was also followed for the comparison of NK-1 receptor immunoreactivity in sham- and IB4-saporin-treated groups in transverse sections and the labeling of non-peptidergic afferents in horizontal sections, however in these two cases IB4 was conjugated to AlexaFluor 568 (Molecular Probes) instead of AlexaFluor 647.

To examine the primary afferent populations in the spinal dorsal horn, sections were processed as described above except that they were incubated for 48 h with IB4 conjugated to AlexaFluor 568, at 1:200 dilution (Molecular Probes) and rabbit anti-CGRP, at 1:2000 dilution (Sigma) followed by incubation with goat anti-rabbit AlexaFluor 488 (Molecular Probes).

Brainstem sections of the injection site were incubated with anti-CTb antibody (List Biological) followed by biotinylated donkey anti-goat IgG (Jackson, West Grove, PA, USA) and streptavidin conjugated to AlexaFluor 568 (Molecular Probes). All sections were mounted and coverslipped as described above.

### Antibody specificity

As controls for immunocytochemistry, some sections were processed by omitting the primary antibodies or by pre-absorption with the peptide used to generate the antibody, in both cases resulting in a complete loss of immunoreactivity.

The goat anti-CTb antibody (List Biological; 703, lot 7032A5) was raised against purified CTb and its specificity was demonstrated by the lack of any staining in animals not injected with CTb. The rabbit anti-NK-1r antibody (Sigma; S8305, lot 084K4845) was generated against a synthetic peptide corresponding to amino acids 393–407 of the C-terminus region of the rat NK-1r conjugated to KLH as the immunogen and purified by ion-exchange chromatography. In Western blots from rat brain, the antibody was found to label only a specific band at 46 kDa, whose staining is specifically inhibited by incubation with the blocking peptide (data supplied by the manufacturer). Furthermore, it was shown that it does not produce any staining in NK-1r knockout mice, although it recognizes the receptor in wild type mice [41]. The rabbit anti-CGRP antibody (Sigma; C8198, lot 070M4835) was generated against synthetic rat CGRP conjugated to KLH as the immunogen. Using dot-blot immunoassay, the antibody was found to recognize rat CGRP conjugated to bovine serum albumin (BSA); it only shows cross-reactivity with CGRP (human) and β-CGRP (human) (data supplied by the manufacturer). Specific staining was abolished by pre-incubating the antiserum with rat CGRP. This antibody was previously used by us as a marker for peptidergic fibers in rat skin, dorsal root ganglia and spinal cord [25, 42–44].

### Morphological characterization and quantification of lamina I neurons

Our criteria of identification and quantification of lamina I neurons have been described extensively in previous publications from our laboratory (see e.g. [24]). In brief, in the current study, six serial, 50 µm-thick horizontal sections were cut from the dorsal part of the L4–L5 spinal segments. Six rats were used per group. Sections were examined under a PlanFluotar 40× oil immersion objective on a Zeiss Axioplan 2e imaging fluorescence microscope. Only neurons with visible nuclei, ipsilateral to the injection side, and with the cell body entirely located within the plane of the section, as assessed with the fine focus of the microscope, were included in our quantifications. Lamina I neurons were classified according to the shape of their cell body and their dendritic arborization in the horizontal plane. Fusiform neurons have two primary dendrites with one arising from each end of an elongated, spindle-shaped soma. Multipolar neurons have an irregularly-shaped cell body with four or more primary dendrites arising from the cell body. Pyramidal neurons have a triangularly-shaped soma with three primary dendrites arising from each of the cell body’s corners, in some cases, a fourth primary dendrite, oriented toward the white matter, was visible by confocal reconstruction or by adjusting the fine focus of a conventional fluorescence microscope. Neurons were classified as “unclassified” if they exhibited features transitional between any of these types, as they did not meet the required criteria.

To obtain images for the illustrations and to confirm the data obtained with conventional fluorescence microscopy, some sections were examined using a Zeiss LSM 510 confocal scanning laser microscope, using a multi-track scanning method and appropriate filters for the separate detections of AlexaFluor 488, AlexaFluor 568 or Rhodamine Red X and AlexaFluor 647. Low magnification images represent single optical section, whereas images of individual neurons represent serial optical sections obtained along the z-axis (z-stacks), using a 63× plan-apochromatic oil-immersion objective.

### Quantitative analysis of CGRP immunolabeling and IB4-binding

Four sham animals and 5 IB4-SAP animals were used for the quantitative analysis. For quantification IB4-positive and CGRP-ir varicosities, single-plane confocal images were obtained in the confocal microscope using the 63× objective, equidistantly from the lateral and medial limits of the dorsal horn. This region corresponded to an area of maximum depletion of IB4 binding ipsilateral to the IB4-SAP injection. The images, originally in the Zeiss file format, were exported to TIFF and quantified using the ImageJ software. For each animal, 8–11 spinal cord cross sections were used, and two images (one from the ipsi- and the other from contralateral side) were obtained per section. In each image, a rectangle of 125 × 110 µm and 125 × 100 µm for IB4 and CGRP labeling, respectively, was placed with longer axis centered on the middle third of lamina II (for IB4) or with the dorsal longer side of the rectangle at on the white matter-lamina I border (for CGRP). To compensate overlapping or clustered varicosities, a correction was performed by the ImageJ software. The average number of varicosities per section’s side (ipsilateral or contralateral), per µm^2^, for each animal was calculated in the two conditions (Sham or IB4-SAP) ± SEM.

### Statistics

Two-way ANOVA followed by Bonferroni correction were applied to compare differences in pain-related behavior at each time point between the sham and experimental groups. One-way analyses of variance (ANOVA) was applied to compare the differences between sham and experimental groups within each neuronal population at the 21 day time point post-IB4-saporin injection. To compare differences in densities of IB4 or CGRP-labeled varicosities, t-tests were used. Values were expressed as mean ± SEM. The significance level was set at P < 0.05. All data were analyzed using GraphPad Prism 5 for Windows (GraphPad Software, San Diego, CA, USA).

### Figure preparation

All immunofluorescence images were obtained with the confocal microscope. They were saved in the Zeiss LSM format, exported as TIFF files and prepared for publication using Adobe Photoshop 7.0 (San Jose, CA, USA). The original images were optimized for brightness and contrast, and pseudo colors were assigned to the markers (green to NK-1r and CGRP and red to IB4 and CtB), to ensure uniformity throughout the paper, but no other image manipulation was done.
